# Profound reduction of HIV-1 reservoir cells over 3 decades of antiretroviral therapy started in early infancy

**DOI:** 10.1172/jci.insight.186550

**Published:** 2024-11-14

**Authors:** Liliana C. Vela, Leah Carrere, Chloe Naasz, Sruthi Kalavacherla, Toong Seng Tan, Lesley de Armas, Ce Gao, Xu G. Yu, Savita G. Pahwa, Katherine Luzuriaga, Mathias Lichterfeld

**Affiliations:** 1 Ragon Institute of MGH, MIT and Harvard, Cambridge, Massachusetts, USA.; 2 Infectious Disease Division, Brigham and Women’s Hospital, Boston, Massachusetts, USA.; 3 Department of Microbiology and Immunology, University of Miami Miller School of Medicine, Miami, Florida, USA.; 4 Program in Molecular Medicine, University of Massachusetts Chan School of Medicine, Worcester, Massachusetts, USA.

**Keywords:** AIDS/HIV, Virology, Adaptive immunity, T cells

## Abstract

HIV-1 reservoir cells persist indefinitely during suppressive antiretroviral therapy (ART) in individuals who acquire infection in adulthood, but little is known about the longitudinal evolution of viral reservoir cells during long-term ART started during early infancy. We studied 2 fraternal twins who acquired HIV-1 perinatally, started ART at week 10 after birth and remained on ART for 28 years. We observed that the frequency of genome-intact proviruses, determined by single-genome near–full-length proviral sequencing, declined by approximately 4,000- to 13,000-fold during this period, indicating enhanced decay rates of intact proviruses even after adjusting for dilution effects from somatic growth. Despite analyzing more than one billion PBMC after 28 years of ART in each participant, no intact proviruses were detected in 1 participant, and 1 intact provirus was isolated in the other. The longitudinal decline of defective proviruses in the 2 participants was more similar to proviral decay kinetics reported in individuals who started ART during adulthood; moreover, clonal sequence clusters were readily detectable for defective proviruses but not for intact proviruses after 28 years of ART in the 2 twins. Together, these data suggest decreased long-term stability and increased immunological vulnerability of intact proviruses during long-term ART started in early infancy.

## Abstract 2

Our longitudinal analysis of two fraternal twins living with HIV who started antiretroviral therapy as infants suggests that early treatment may enhance viral control over time.

## Introduction

Although perinatal HIV-1 infection is rare in the Unites States, current estimates suggest that globally, approximately 100,000–150,000 new cases occurred in 2022 (1), reflecting a need for improved interventions for the prevention and treatment of HIV-1 in neonates, infants, and young children. In the absence of antiretroviral therapy (ART), children living with HIV-1 experience more rapid disease progression than adults (2, 3) and frequently develop complications in growth and development (4). Thus, initiation of ART as soon as possible, ideally within hours or days after diagnosis, is recommended for all infants and children living with HIV-1 (5). However, treatment initiation during even the earliest stages of HIV-1 infection cannot prevent the establishment of a long-lasting pool of infected cells that persist indefinitely and can fuel viral rebound when suppressive antiviral therapy is discontinued (6–8). These cells, frequently termed “HIV-1 reservoir cells,” contain chromosomally integrated viral DNA sequences that typically express limited viral gene transcripts, allowing them to avoid host immune recognition (9, 10). Nevertheless, recent data suggest that the human immune system is not powerless against such reservoir cells; in fact, a series of recent data suggest that proviruses harbored by these reservoir cells can be subject to selection pressure, likely mediated by host immune responses that can target at least some of these cells (11), presumably due to residual viral transcription that occurs in a certain number of reservoir cells and exposes them to immune recognition (11). Footprints of such immune selection activities are most obvious when proviral integration sites are analyzed (12, 13) and/or when phenotypic signatures of virally infected cells are evaluated (14), but the immune mechanisms that can target infected cells and drive immune selection are not well defined at present. However, it is clear that immune activity against viral reservoir cells can vary profoundly among different individuals, with factors such as age, sex (15), and immunogenetic characteristics likely to play an important role. How the pediatric immune system engages and targets HIV-1 reservoir cells is an area of active investigation. In the present study, we used highly sensitive techniques to characterize the persisting viral reservoirs of fraternal twins with intrapartum HIV-1 infection who started ART at 10 weeks of age and remained on continuous antiviral therapy for 28 years.

## Results

### Clinical histories of 2 fraternal twins treated with ART for 28 years.

We focused our analyses on fraternal twins, P-1042 and P-1043, who acquired HIV-1 perinatally. The mother did not engage in prenatal care and was not tested prenatally for HIV-1; she did not receive antiviral agents during pregnancy nor during delivery. Both infants were treated with zidovudine prophylaxis up to 6 weeks of age. Initial HIV-1 nucleic acid testing and cultures were negative. At 1.2 months of age, HIV-1 DNA was detected by qualitative assays in P-1042’s PBMC; P-1043 was negative for viral DNA at that time, but plasma viral RNA was positive with 789 copies/mL. Multiple blood samples were subsequently positive for HIV nucleic acids, with plasma HIV-1 RNA as high as 399,943 (P-1042) and 370,279 (P-1043) copies/mL, consistent with intrapartum infection (16). The twins were started on ART at 10 weeks of age and remained on continuous suppressive treatment for the subsequent 28 years. Plasma viral loads became undetectable at 8 months (P-1042) and 4 months (P-1043) of age. Longitudinal plasma viral loads, proportions of CD4 T cells, and antiretroviral drug exposure are summarized in Supplemental Figure 1 and Supplemental Table 1 (supplemental material available online with this article; https://doi.org/10.1172/jci.insight.186550DS1). P-1042 is a male patient and had a brief period of detectable viremia (RNA 1117 copies/mL) at 18 months, but there were no recorded episodes of detectable plasma viremia using commercial PCR assays during the subsequent time period of observation. P-1043 is a female patient and experienced no documented viral blips during the entire duration of antiviral therapy other than 1 RNA value of 42 copies/mL at 23 years of age. Neither twin expressed HLA class I alleles previously associated with spontaneous control of HIV-1 (17). Longitudinal PBMC samples were available from 6 months after ART initiation and from a number of follow-up time points over almost 3 decades (28 years) of antiviral therapy.

### Proviral sequencing analysis.

**Figure 1:**
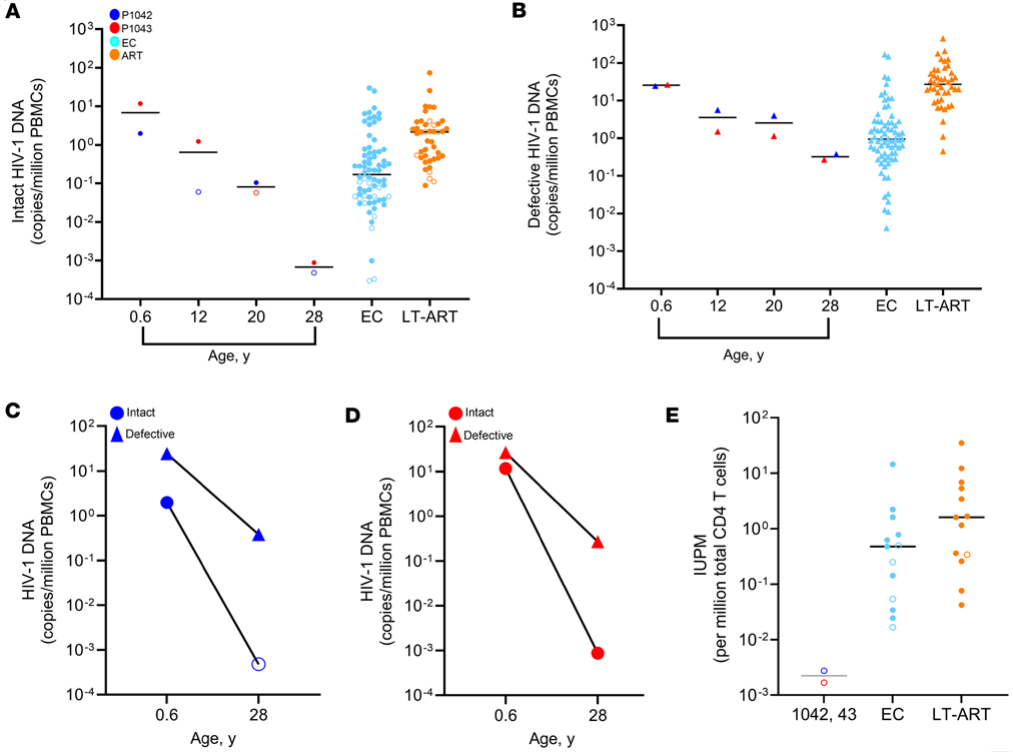
Longitudinal evolution of HIV-1 reservoir cells in 2 fraternal twins with vertical HIV-1 infection over 28 years of antiretroviral therapy. (**A** and **B**) Frequency of intact (**A**) and defective (**B**) proviruses in P-1042 and P-1043 at indicated time points of age. Corresponding data from ART-treated adults (ART, median duration of ART of 12.84 years) and from elite controllers (EC) are shown for comparison. (**C** and **D**) Fold changes between intact and defective HIV-1 proviruses measured in samples collected at 6 months and 28 years of age in P-1042 and P-1043. (**E**) Infectious units per million CD4 T cells, measured by in vitro viral outgrowth assays, in the 2 twins and in the reference cohorts of EC and adults on ART. Open symbols: indicate measurements at the limit of detection, indicated as the number of cells analyzed without target identification.

To profile the evolution of viral reservoir cells, we used near full-length, single-genome next-generation proviral sequencing (18, 19), permitting us to identify the frequency of genome-intact proviruses and of defective proviruses exhibiting structural variations precluding viral replication. To avoid contamination of sequences from both study participants that derived from the same maternal source virus, experiments related to each twin were conducted at physically separated research buildings. Six months after treatment initiation, a total of 11 and 48 intact proviruses were detected in P-1042 and P-1043, respectively, resulting in a relative frequency of 2.17 and 11.90 genome-intact DNA copies per million PBMC at this time (Figure 1, A and B, and Supplemental Table 2). After 12 years of antiviral therapy, no intact proviruses were detected in 8.4 million PBMC from P-1042, while defective proviruses were readily detectable; 9 intact proviruses, most of which were part of a large clonal cluster, were detected in 7.3 million PBMC from P-1043 at this time point (Figure 1, A and B). Eight years later, after a total of 20 years of continuous treatment, 1 intact provirus was observed in 9.6 million PBMC from P-1042 and no intact proviruses were detected in 8.7 million PBMC from P-1043. However, we acknowledge that the quantification of intact proviruses from these two time points was less precise, given the small reservoir size and the limited number of PBMC available from these follow-up time periods. After 28 years of continuous ART, large numbers of PBMC were available from leukapheresis procedures from both twins. At this time point, no intact proviruses were detected in P-1042 after analyzing a total of 976 million PBMC and 72.9 million isolated CD4 T cells. In P-1043, one intact provirus was observed at that time after analyzing 1,080 million PBMC and 62 million isolated CD4 T cells (Figure 1, A and B, and Supplemental Table 2). Together, this analysis demonstrated that the frequencies of intact proviruses after 28 years of continuous antiviral therapy were approximately 6,716-fold lower compared with a group of adult people living with HIV-1 who had remained on ART for a median of 12.84 years analyzed previously (20) and approximately 2,580-fold lower compared with a group of elite controllers described in our prior work (20) (Figure 1, A and B, and Supplemental Table 3). A total of 580 and 439 defective proviruses were detected in P-1042 and P-1043, respectively. The majority of these defective proviral sequences were isolated from the final analysis time point after 28 years of ART when leukapheresis samples were available. At this time, the frequencies of defective proviruses in the 2 twins were approximately 24- and 160-fold lower compared with elite controllers and ART-treated persons, respectively. Corresponding to these findings, we noted that the frequency of intact proviruses in P-1042 declined by approximately 4,140-fold after 28 years of antiviral therapy relative to the baseline level obtained 6 months after ART initiation, while defective proviruses declined by 64-fold over the same time period. In P-1043, intact proviruses declined by 13,345-fold, and defective proviruses declined by 98-fold over the 28-year observation period, relative to the baseline levels (Figure 1, C and D). To complement these findings, we conducted traditional in vitro viral outgrowth assays using 298 million CD4 T cells from P-1043 and 181 million CD4 T cells from P-1042 collected by leukapheresis from the 28-year time point; however, no viral outgrowth was recorded in any of these experiments. In contrast, viral outgrowth was readily detectable in almost all persons from the control cohort of elite controllers and of ART-treated adults using the identical experimental protocol (Figure 1E).

### Clonality and integration site analysis.

**Figure 2:**
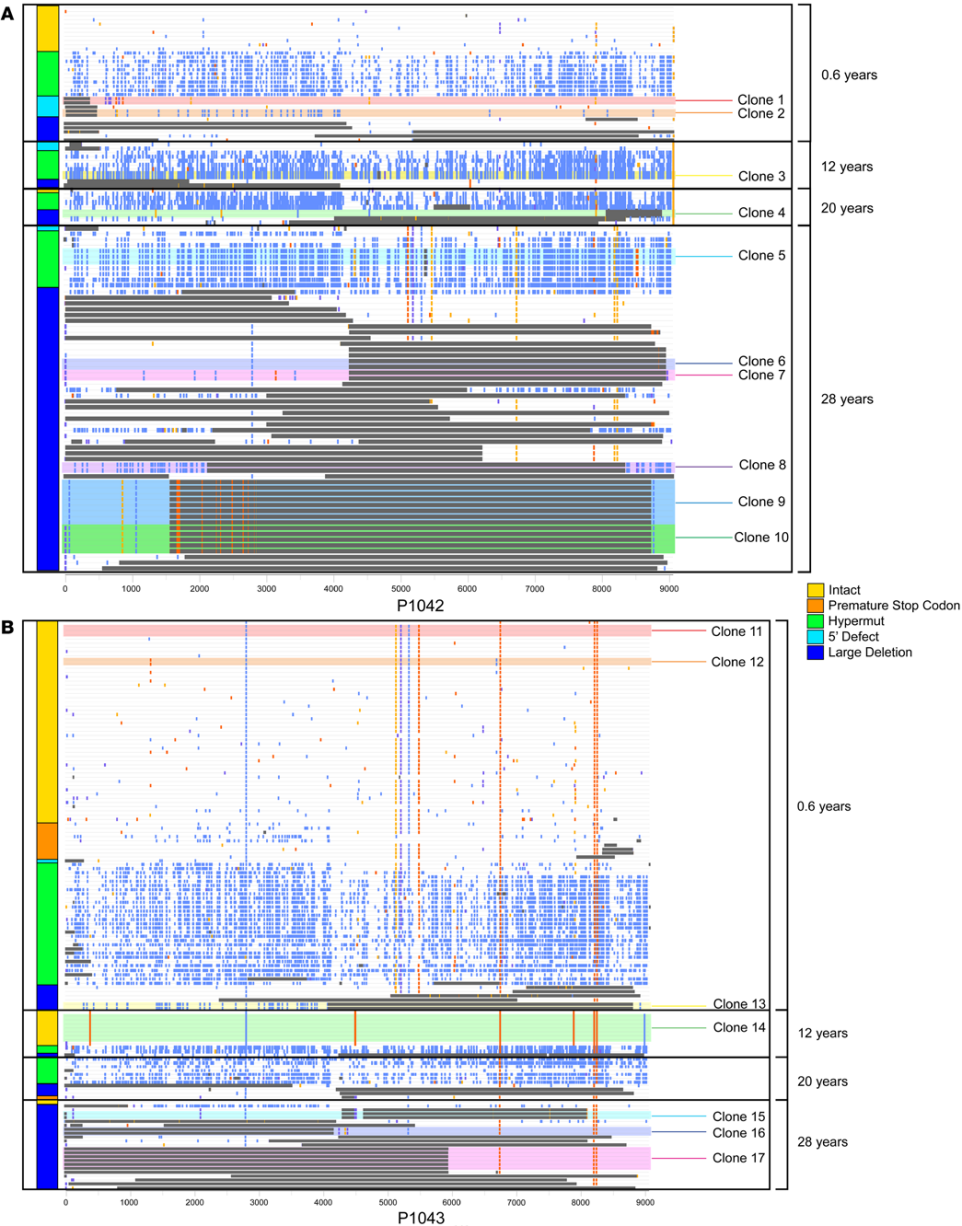
Proviral landscape in the 2 fraternal twins over two 28 years of antiretroviral therapy. (**A** and **B**) Highlighter plots reflect all proviral sequences isolated from P-1042 and P-1043 at indicated time points; each horizontal line represents 1 provirus. The *y* axes indicate the age at which samples were obtained for study. Color coding reflects proviral sequence classification. Clusters of sequences-identical proviral clones are highlighted.

**Figure 3:**
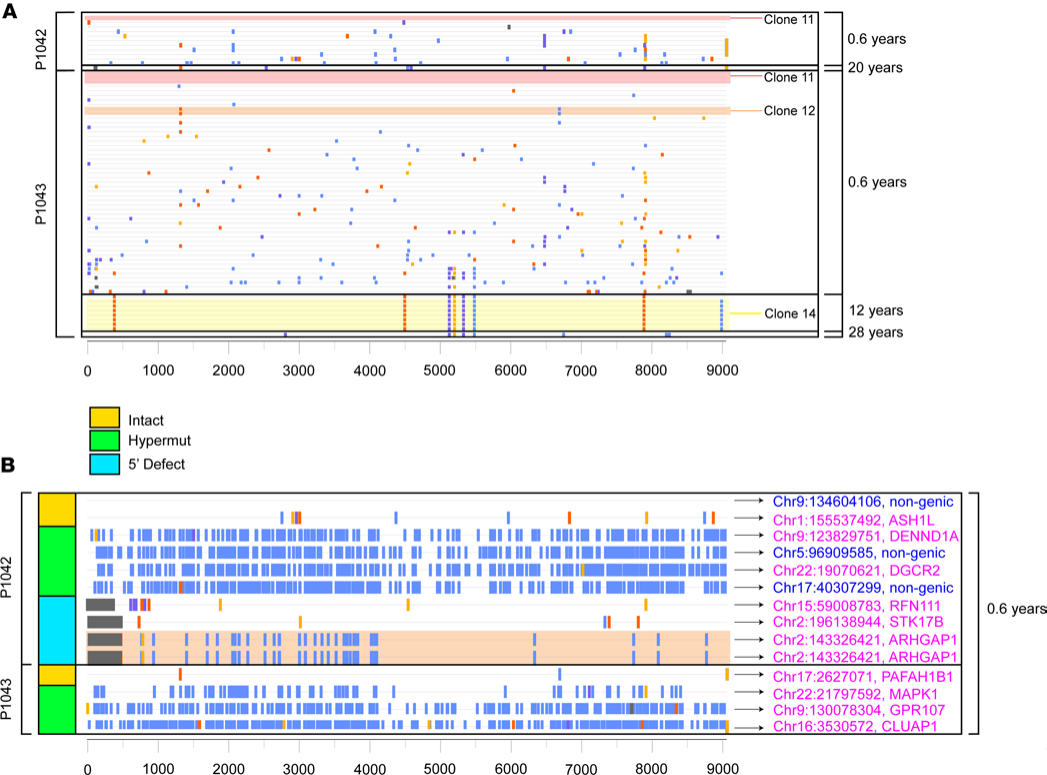
Proviral clonality and integration sites in the study participants. (**A**) Landscape of genome-intact proviruses isolated from the 2 study participants at indicated time points. Clusters of sequence-identical clones are highlighted. (**B**) Proviral integration site of proviruses isolated at 8 months after birth (5 months after ART initiation). The *y* axes in **A** and **B** indicate age at which samples were obtained for study.

For a deeper analysis of the viral reservoir landscape in both twins, we aligned all sequencing reads from each study participant, permitting us to identify sequence-identical clones of proviruses that reflect in vivo proliferation of infected cells (19, 21–24). In both twins, we noted that clonal genome-intact proviral sequences were already detectable at 6 months after treatment initiation (clones 1, 2, 11, 12, 13), although at relatively small frequencies (Figure 2). At subsequent follow-up time points, multiple clonal clusters of defective proviruses were noted in both twins; however, no clonal clusters of intact proviruses were detected in P-1042 during follow-up assessments. In P-1043, a larger clone of intact proviruses was observed after 12 years of ART (clone 14; Figure 3A); however, these or other clonal genome-intact clonal sequences were not detected in the large numbers of cells analyzed at 28 years in twin P-1043. Of note, 1 intact sequence identified at 8 months of age (after 6 months of ART) in P-1042 was completely identical to an intact provirus in P-1043 detected at the same time point (clone 11, Figure 3A); due to the physical separation of the sequencing work at two distinct geographic locations, this is unlikely to represent PCR contamination but rather reflects the identification of an identical maternal founder virus transmitted to both infants. As an additional investigative modality, we analyzed chromosomal integration sites in some proviruses identified during the earliest time point of analysis, using a previously described protocol (23); these studies mostly identified integration sites in genic locations that are preferred by the viral integration machinery (25) (Figure 3B). The low frequency of intact proviruses at later stages of ART precluded the identification of chromosomal locations for proviruses from these time points.

### Proliferation assays.

**Figure 4:**
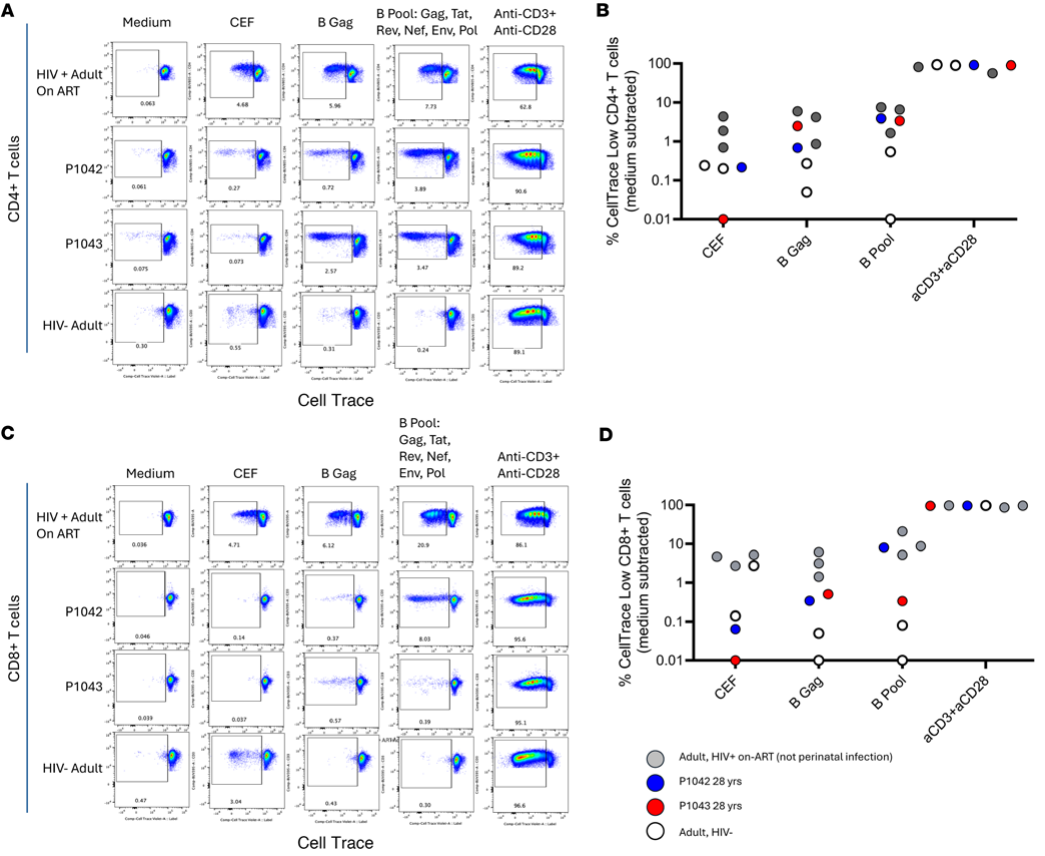
HIV-specific T cell responses after 28 years of ART started in infancy. (**A** and **C**) CD4^+^ (**A**) and CD8^+^ (**C**) T cell proliferation in response to stimulation with indicated clade B HIV-1 antigens or with CMV/Epstein-Barr virus/influenza virus (CEF) antigens in the 2 study participants, in ART-suppressed adults with HIV-1, and in adults without HIV-1. (**B** and **D**) Cumulative T cell proliferation data in responses to indicated antigens in P1042, P1043, and adults with or without HIV-1.

To complement the analysis of viral reservoir cells, we investigated adaptive immune responses to HIV-1 in the 2 twins after pharmacological suppression of viral replication for 28 years. Western blot analysis demonstrated no detectable HIV-specific antibodies in study participant P-1042 after 28 years of ART, while in P-1043, an incomplete HIV Western blot with a single p24-specific antibody reaction was noted at that time. Prior assessments of HIV-specific T cell responses using ELISpot assays failed to identify cellular immune responses in either twin after initiation of ART (26). To extend these findings, frequencies of proliferating CD4 and CD8 T cells were determined in response to a peptide pool containing consensus subtype B sequences encompassing multiple HIV-1 antigens as well as a pool containing overlapping Gag peptides, using cells from the final analysis time point. P-1042 had a more robust CD4 T cell response to the combined HIV-1 antigen pool compared with the Gag pool, suggesting the presence of non–Gag-specific CD4 T cells, while P-1043 had comparable proliferation to the antigen pool and Gag (Figure 4A). We compared the twins’ HIV-1 specific T cell responses with adult PBMC donors without HIV-1 (*n* = 2) and ART-suppressed people who acquired HIV-1 in adulthood (*n* = 3). Proliferation in response to HIV-1 peptides was observed in T cells from individuals living with HIV but not from HIV-negative study participants (Figure 4, A and B). Overall, the twins’ CD4 T cells exhibited similar magnitudes of proliferation as adults who acquired HIV-1 horizontally. The CD8 T cell response to Gag was much lower in the twins compared with adults living with HIV-1; however, it was still detectable with frequencies greater than 5 times the medium control (Figure 4C). Again, P-1042 showed robust CD8 T cell proliferation to the consensus subtype B antigen pool comparable with adults with HIV-1, which was not observed in P-1043 (Figure 4D). Together, these results demonstrate the high durability and persistence of HIV-1–specific memory T cell responses despite multiple decades of suppressive ART.

## Discussion

We describe a detailed analysis of the proviral reservoir in 2 fraternal twins with perinatal HIV-1 infection who started ART 10 weeks after birth and then remained on continuous ART for about 28 years. While the frequency of intact proviruses in both study participants at baseline (6 months after ART initiation) was quite similar to previously reported data from adults (19), our results demonstrate a number of distinct viral reservoir features during long-term ART started in early infancy. First, the frequencies of intact proviral sequences after 28 years of antiviral therapy were extremely low, with no detectable genome-intact proviral sequences in more than 1 billion analyzed cells in 1 twin (P-1042) and only 1 genome-intact sequence in more than 1 billion analyzed cells in the other twin (P-1043). These observations represent a profound contrast to the vast majority of individuals who acquired HIV-1 in adulthood, in whom intact proviruses are typically readily detectable in 10 million to 20 million PBMC using full-genome sequencing (20), even when exposed to very long periods of ART (27, 28). Second, we found no evidence for clonal proliferation of cells harboring genome-intact proviruses after 28 years of therapy. On the contrary, large clones of intact proviruses typically dominate the proviral landscape in many long-term ART–treated adults living with HIV-1 and may drive the expansion of the viral reservoir cell pool during prolonged periods of ART in adults (13, 18, 28–30). Corresponding to these observations, we noted a profound decline in the frequency of intact proviruses over 28 years, by a factor of 1:4,104 (P-1042) and 1:13,345 (P-1043). In contrast, the half-life of intact proviruses following HIV-1 transmission in adulthood has been reported to reach approximately 4 years during the first 7 years after ART initiation and approximately 18 years afterward (31), which would result in a calculated decline of intact proviruses by approximately 10-fold over 28 years. Together, these data raise the possibility that ART initiation in neonates or during early infancy is associated with a distinct evolutionary trajectory of viral reservoir cells.

Several factors may contribute to such an atypical viral reservoir evolution during infancy, childhood, and adolescence. Somatic growth may be an important factor that could lead to dilution effects on viral reservoir cells during the first decades of life and may partially explain the very low frequencies of intact proviruses. However, when normalizing the decline of intact proviruses by a factor of 20 (assuming a 20-fold increase in body weight over the initial 3 decades of life), the reduction of intact proviruses over 28 years would reach a factor of approximately 200- to 650-fold, which still profoundly exceeds the approximately 10-fold decline that would be expected to occur over 28 years of ART in adults based on prior calculations of the viral half-life (31). Assuming the same dilution factors of 20 for defective proviruses, the normalized reduction of defective proviruses would be approximately 3- to 5-fold in the 2 twins, which represents a more limited difference to the calculated approximately 1.5- to 2-fold reduction of defective proviruses that would be expected to occur over 28 years of continuous ART in adults, based on prior studies demonstrating a half-life of defective proviruses of 17 years during the first 7 years of ART and of 45 years afterward in adulthood (31). We also considered the possibility that intact proviruses may have declined more rapidly in these 2 twins due to the initiation of ART early after infection, given that timing of treatment commencement can influence the kinetics of viral reservoir cell decline (6). However, we have previously described an adult who initiated early ART (stage 4 of primary infection; participant LT-02) (27) in whom the frequency of intact proviruses was 2 per million PBMC after approximately 20 years of ART; in a second adult who initiated ART early (stage 4 of primary infection), we noted a frequency of 1 intact provirus per million PBMC after 20 years of ART. Together, these data suggest that treatment initiation in early infancy, followed by prolonged ART, is associated with an accelerated clearance of genome-intact HIV-1 sequences that cannot be exclusively explained by the dilution of infected cells due to somatic growth or early treatment initiation. Instead, we hypothesize that the profound reduction of genome-intact HIV-1 sequences after ART initiation in early infancy in the 2 twins described here may be related to more effective immunological targeting and elimination of cells harboring such genome-intact viral sequences by the pediatric immune system. How exactly the pediatric immune system may have improved abilities to clear viral reservoir cells is unknown at present but may involve distinct functional profiles of innate and adaptive effector cells, altered cytokine milieus, or specific changes in global immune regulation and activation occurring in the developing immune system; moreover, the fact that HIV-1 reservoir cells seeded in early infancy are likely to include naive CD4 T cells may differentially affect viral persistence mechanisms. Future studies will be necessary to evaluate the longitudinal evolution of viral reservoir cells in neonates, infants, children, and adolescents to further explore effects of pediatric immune cells on the viral reservoir cell pool. Of note, improved antiviral activity of the pediatric immune system against viral infections has been documented in specific viral illnesses (32), arguably most definitively in the context of COVID-19 infection (33).

This study has several limitations. First, all studies were conducted with cells from the peripheral blood, and the longitudinal changes in lymphoid tissue reservoirs in the 2 study persons remain unknown at present. Second, while transmission of the same founder virus to both study participants reduced stochastic analysis effects related to viral sequence diversity, future studies with a range of different viral strains will be necessary to better evaluate effects of the pediatric immune system on HIV-1 persistence. Moreover, the types of immune responses that are responsible for targeting and reducing HIV-1 reservoir cells in our study participants are unknown. Proliferation assays identified HIV-1–specific memory T cells that persisted over 28 years of ART in both individuals. However, we do not claim that these types of immune responses are etiologically related to viral reservoir dynamics; detailed immune profiling experiments, including those involving single-cell immunophenotypic assays of HIV-infected target cells and immune effector cells, will be needed to address this further. We also acknowledge that modeling of longitudinal decay kinetics of intact proviruses is complex and not uncontroversial (34), making comparisons between viral reservoir dynamics in our study participants and those of adult reference cohorts (31) more difficult. Finally, the clinical significance of extremely low reservoirs of genome-intact proviruses in our study participants remains unknown at present; future analytical treatment interruptions may be considered to address this. Due to the relatively high frequencies of perinatal HIV-1 infection that occurred more than 20 years ago when effective measures to prevent mother-to-child transmission were not available in many parts of the world, there is now a growing generation of teenagers and young adults who initiated ART in early infancy and have remained on ART for several decades. Understanding how the pediatric immune system recognizes, targets, and eliminates HIV reservoir cells in these persons, therefore, remains an important scientific question with marked public health implications.

## Methods

### Sex as a biological variable.

Our study focuses on the analysis of 2 fraternal twins; 1 is male, and 1 is female. Sex was not considered as a variable for outcome parameters.

### Study participants.

Study participants living with HIV-1 were recruited and followed at the University of Massachusetts Chan School in Worcester, Massachusetts, USA. Study participants from reference cohorts were recruited at Massachusetts General Hospital (Boston, Massachusetts USA), as described in a previous study (20).

### Cell samples.

PBMCs were collected at 0.6, 12, 20, and 28 years after ART initiation by standard blood draw or by leukapheresis and isolated using Ficoll-Paque density centrifugation (at 400 x g). PBMCs were viably frozen in 90%–95% FBS and 5%–10% dimethylsulfoxide.

### Quantitative viral outgrowth assay (qVOA).

Immunomagnetic enrichment of CD4 T cells was performed using the EasySep Human CD4^+^T cell isolation Kit (Stemcell Technologies, 17952) using the manufacturer’s protocol. Large-scale quantitative viral outgrowth measurements on cells from the participants were performed as described (20), with a luciferase reporter assay used to detect viral outgrowth.

### Full-length individual proviral sequencing.

DNA was extracted from PBMCs by using commercial kits (DNeasy Blood & Tissue Kit, QIAGEN). Total HIV-1 DNA and cell numbers were quantified with Droplet Digital PCR (ddPCR, Bio-Rad), using primers and probes that have been described previously (18). DNA was diluted to single-genome levels based on Poisson distribution statistics and droplet digital PCR (ddPCR) results and was then subjected to single-genome near-full-length HIV-1 amplification, as previously described. Individual amplification products were sequenced on the Illumina MiSeq platform. Resulting short reads were de novo assembled and aligned to HXB2. Intact and defective proviral sequences were distinguished using an automated pipeline written in Python code (https://github.com/ BWH-Lichterfeld-Lab/Intactness-Pipeline) (commit id: 0c59371). The presence or absence of APOBEC-3G/3F–associated hypermutations was determined using the Los Alamos HIV Sequence Database Hypermut 2.0 program (https://www.hiv.lanl.gov/content/sequence/HYPERMUT/hypermut.html). Viral sequences were considered clonal if they had completely identical sequences. For combined integration site analysis and proviral sequencing, near full-length viral amplification was preceded by a whole genome amplification step using phi29 polymerase (35).

### Integration site analysis.

Integration sites were obtained using integration site loop amplification (ISLA), using a previously described protocol (36). PCR products of the ISLA reaction were subjected to next-generation sequencing using Illumina MiSeq. MiSeq paired-end FASTQ files were demultiplexed; small reads (142 bp) were then aligned simultaneously to the human reference genome GRCh38 and HIV-1 reference genome HXB2 using bwa-mem. Biocomputational identification of integration sites was performed according to previously described procedures. The final list of integration sites and corresponding chromosomal annotations was obtained using the UCSC Genome Browser (www.genome.ucsc.edu).

### Western blots.

HIV Western blots were carried using standard commercial assays.

### Proliferation assays.

Cryopreserved PBMC were thawed and rested for 3 hours before labeling with CellTrace Violet (CTV) proliferation tracking dye at a dilution of 1:100 CTV per 1 million to 3 million cells/mL according to the manufacturers protocol. Labeled PBMC were resuspended at 4 million cells/mL in complete RPMI medium (with 10% FBS and penicillin/streptomycin, Thermo Fisher Scientific). Five-day stimulation cultures were set up in 24-well flat-bottom plates containing 500 μL of cell preparation and HIV-1 Subtype B Consensus peptide pools (BEI Resources). Gag Peptide pool was used at 1 μg/mL, and the Pool containing Tat, Env, Pol, Nef, Gag, and Rev peptide pools had a final concentration of 1 μg/mL (with each antigen at 0.166 μg/mL). For experimental controls cells were stimulated with cytomegalovirus, Epstein-Barr virus, influenza virus (CEF) peptide pool as an alternate antigenic stimulation (2 μg/mL, BEI Resources), anti-CD3 (clone HIT3a, 0.01 μg/mL, BD Biosciences) to induce T cell proliferation, or DMSO (0.5%) as a negative control. All wells received anti-CD28 antibodies (1 μg/mL, clone L293, BD Biosciences) for costimulation.

Cultures were incubated for 5 days at 37°C in 5% CO_2_. On day 3, media were changed by removing 500 μL of media and replenishing with fresh complete RPMI. On day 5, cells were harvested and stained with Live/Dead Aqua followed by staining for CD45 (clone 30-F11), CD3 (clone SK7), CD4 (clone SK3), CD8 (clone SK1), and CD56 (NCAM16.2) acquired on a Cytek Aurora flow cytometer. Manual gating was performed using unstained and FMO controls using FlowJo Software. Frequencies of proliferating cells were calculated based on gating of CellTrace dye (Thermo Fisher Scientific) in the medium (negative control sample). Upstream gating of proliferating T cells is shown in Supplemental Figure 2.

### Study approval.

The IRBs of University of Massachusetts Chan School and of the Ragon Institute approved these studies; study participants and/or their guardians gave written informed consent.

### Data availability.

Viral sequencing data were deposited in GenBank (accession nos. PQ511494 - PQ511753). Values for all data points in graphs are reported in the Supporting Data Values file.

## Author contributions

Clinical follow-up and biological specimen collection were contributed by KL. Analysis of viral DNA was contributed by LCV and SK. Viral outgrowth assays were contributed by LCV and TST. Integration site analysis was contributed by LCV, LC, SK, and CN. Bioinformatics analysis was contributed by CG. Flow cytometry and proliferation assays were contributed by LDA and SGP. Data analysis, interpretation, and presentation were contributed by KL, LCV, SK, LDA, SGP, CG, XGY, and ML. Writing and preparation of manuscript and figures were contributed by KL, LCV, SK, LDA, and ML. Project supervision was contributed by KL, SGP, XGY, and ML.

## Supplementary Material

Supplemental data

Supporting data values

## Version 1 11/14/2024

In-Press Preview

## Version 2 01/09/2025

Electronic publication
