# Supplementary material for: Profound reduction of HIV-1 reservoir cells over 3 decades of antiretroviral therapy started in early infancy
Source: JCI Insight. 2024 Nov 14;10(1):e186550. doi: 10.1172/jci.insight.186550 (PMC11721289; doi:10.1172/jci.insight.186550)

Supplemental Figure and Table Legends

Supplemental Table 1: CD4/CD8 T cell ratios for study participants P1042 and P1043.

Supplemental Table 2: Summary of intact and defective HIV-1 sequences isolated from study participants P1042 and P1043 at indicated timepoints.

Supplemental Table 3: Clinical and demographic data of comparison cohorts of ART-treated adults (ART, median duration of ART of 12.84 years) and elite controllers (EC).

Supplemental Figure 1. Longitudinal HIV-1 plasma viral loads and proportions of CD4 T cells in P-1042 and P-1043. Antiretroviral treatment exposure is indicated on x-axis. Diamonds: proportions of CD4 T cells (%); Closed circles: RNA Viral load > LOD; Open circles: RNA Viral load < LOD; Red arrow: Single RNA Viral load > LOD in P-1043 after suppression. Vertical shading indicates timepoints at which samples were analyzed. ZDV, zidovudine; ddI, didanosine; NVP nevirapine; d4T, stavudine; 3TC, lamivudine; TDF, tenofovir disoproxil fumarate; FTC, emtricitabine; ABC, abacavir; ATV/r = Ritonavir-boosted Atazanavir; DTG, dolutegravir

Supplemental Figure 2. Gating scheme for T cell proliferation experiments. Representative counter plots from the negative control condition from representative HIV-negative donor are shown.

Supplemental Table 1

P1042			
Age (years)	CD4%	CD8%	CD4/CD8 ratio
0.6	60	19	3.2
12	45	18	2.5
20	51	21	2.4
28	54	15	3.6
**P1043**			
Age (years)	CD4%	CD8%	CD4/CD8 ratio
0.6	60	17	3.5
12	52	21	2.5
20	54	22	2.5
28	57	22	2.6

Supplemental Table 2

P1042<br>HLA-A*26:08, HLA-A*30:02, HLA-B*18:01, HLA-B*39:01, HLA-C*05:01, HLA-C*12:03								
Timepoint	0.6 years		12 years		20 years		28 years	
Total Cells Assayed (in millions of PBMCs/CD4 T cells)	5.07		8.37		9.57		1,049 (976 million PBMC, 73 million CD4 T cells)	
	Absolute number of sequences	Sequences per million PBMC	Absolute number of sequences	Sequences per million PBMC	Absolute number of sequences	Sequences per million PBMC	Absolute number of sequences	Sequences per million PBMC
Intact sequences	11	2.17	0	0.00	1	0.10	0	0.00
5' Defect sequences	5	0.99	2	0.24	0	0.00	0	0.00
Hypermutated sequences	11	2.17	7	0.84	4	0.42	10	9.53 x 10^{-4}
Sequences with a premature stop codon	0	0.00	0	0.00	0	0.00	1	9.53 x 10^{-4}
Sequences with internal inversion	1	0.20	0	0.00	0	0.00	16	1.53 x 10^{-2}
Sequences with large deletions	111	21.89	40	4.78	34	3.55	371	0.35
Total defective sequences	128	25.24	49	5.62	38	3.97	398	0.38

P1043<br>HLA-A*11:01, HLA-A*26:08, HLA-B*39:01, HLA-B*51:01, HLA-C*05:03, HLA-C*12:03								
Timepoint	0.6 years		12 years		20 years		28 years	
Total Cells Assayed (in millions of PBMCs/CD4 T cells)	4.04		7.31		8.71		1,142 (1080 million PBMC, 62 million CD4 T cells)	
	Absolute number of sequences	Sequences per million PBMC	Absolute number of sequences	Sequences per million PBMC	Absolute number of sequences	Sequences per million PBMC	Absolute number of sequences	Sequences per million PBMC
Intact sequences	48	11.90	9	1.23	0	0.10	1	8.73 x 10^{-4}
5' Defect sequences	1	0.25	0	0.00	0	0.00	0	0.00
Hypermutated sequences	26	6.44	2	0.27	2	0.23	5	4.36 x 10^{-3}
Sequences with a premature stop codon	9	2.23	0	0.00	0	0.00	1	8.73 x 10^{-4}
Sequences with internal inversion	1	0.35	0	0.00	0	0.00	2	1.75x 10^{-3}
Sequences with large deletions	70	17.35	9	1.23	8	0.92	303	0.26
Total defective sequences	107	26.52	11	1.50	10	1.15	311	0.27

Supplemental Table 3

Cohort	Number of Participants	% Male	% Female	Age (years)	CD4 Count/ul (median, range)	Recorded duration of undetectable viremia in years (median, range)	Duration of ART (median, range)
EC	72	81.9	18.1	57 (31-75)	882 (450, 2282)	8.8 (1, 24.2)	N/A
ART	43	69.8	30.2	55 (34, 73)	727 (316, 1657)	8 (1, 19)	12.84 (1.3, 21.3)

Supplemental Figure 1

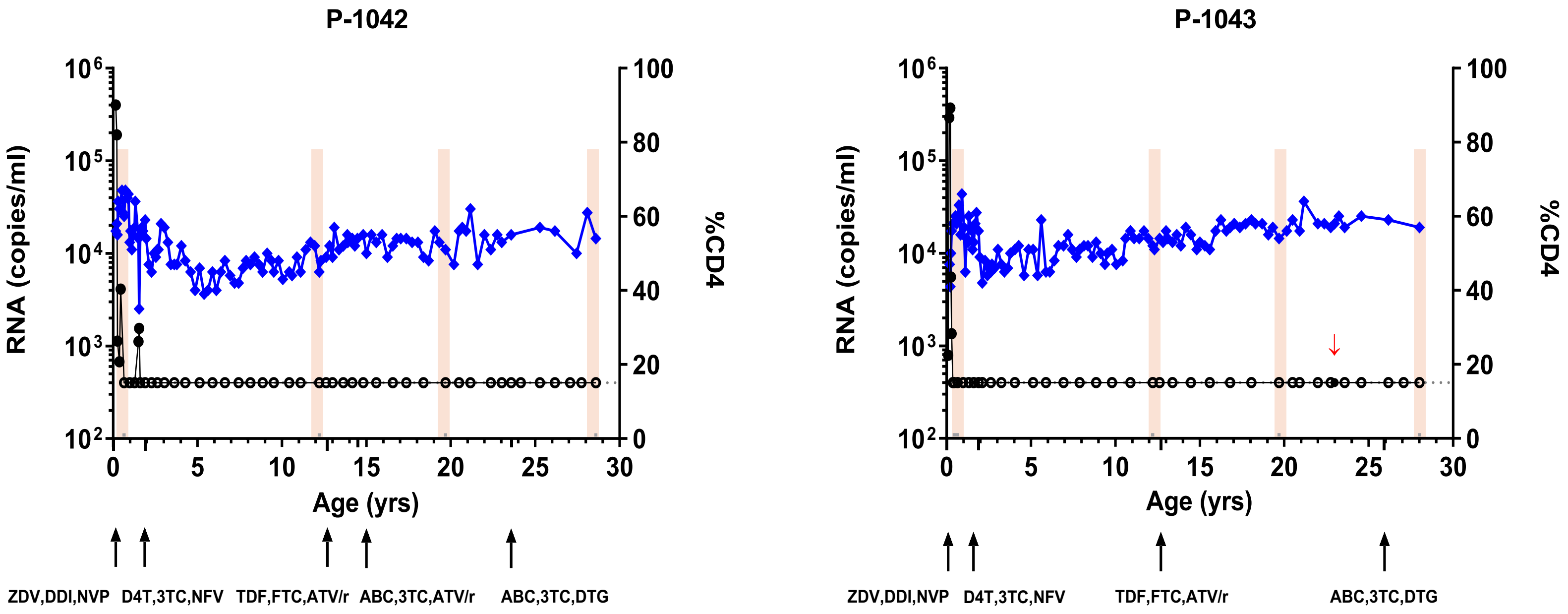

Supplemental Figure 2

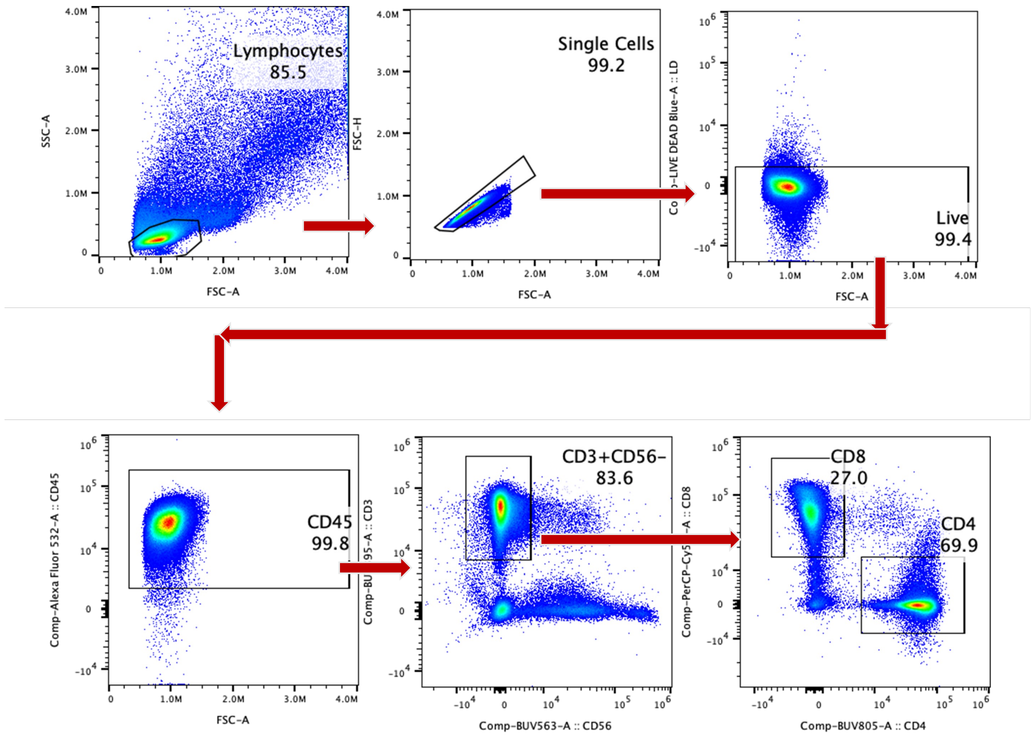

Supplement: Supplemental data [file jciinsight-10-186550-s289.pdf]
